# Is Wushu participation associated with internalizing problem behaviors among Chinese adolescents? A cross-sectional baseline survey

**DOI:** 10.3389/fpubh.2026.1879903

**Published:** 2026-09-14

**Authors:** Minqing Yang, Xiangshan Ge, Defa Zhang

**Affiliations:** 1 School of Physical Education, Suihua University, Suihua, China; 2 School of Foreign Studies, Suzhou University, Suzhou, Anhui, China

**Keywords:** adolescents, anxiety, depression, event category, organizational form, stress, Wushu

## Abstract

**Objective:**

This study examined the cross-sectional associations between Wushu participation and internalizing problem behaviors, including depression, anxiety, and stress, among Chinese primary and secondary school students. It also explored whether organizational forms of participation and Wushu event categories were associated with these mental health indicators.

**Methods:**

A cross-sectional research design was adopted, with a valid sample of 6,642 participants. Questionnaires were used to assess Wushu participation and levels of depression, anxiety, and stress. Demographic information, including gender, grade, age, residence, and parental educational attainment, was also collected. Harman’s single-factor test was conducted to assess common method bias, followed by descriptive statistics and correlation analyses. Multiple linear regression models were used to estimate the adjusted cross-sectional associations of Wushu participation, organizational form, and event category with depression, anxiety, and stress after controlling for selected demographic covariates. Collinearity and residual diagnostics were also performed.

**Results:**

(1) The common method bias test showed that the first common factor explained 26.19% of the total variance, lower than the critical standard of 40%. (2) Wushu participation was significantly negatively correlated with depression (*r* = −0.261), anxiety (*r* = −0.251) and stress (*r* = −0.207) (all *p* < 0.01); depression, anxiety and stress were significantly positively correlated with one another (*r* = 0.423–0.473, all *p* < 0.01). (3) In the adjusted models, Wushu participation was significantly and negatively associated with depression *β* = −0.191, anxiety *β* = −0.166, and stress *β* = −0.156 all *p*<0.001. Organizational form and event category were also associated with the three mental health indicators *p* < 0.05 or *p* < 0.001.

**Conclusion:**

Among Chinese primary and secondary school students, higher levels of Wushu participation were cross-sectionally associated with lower levels of depression, anxiety, and stress. Organizational form and Wushu event category were also associated with these mental health indicators, suggesting that participation context may be relevant when considering the potential public health value of Wushu practice.

## 1 Introduction

Since the beginning of the new century, health problems such as overweight/obesity and poor eyesight among Chinese adolescents have attracted continuous attention, and insufficient physical activity has been regarded as one of the important modifiable risk factors. The World Health Organization (WHO) and guidelines of many countries emphasize that children and adolescents should maintain adequate moderate-to-vigorous physical activity; however, the phenomenon of insufficient compliance is still prevalent globally, with significant differences across different socioeconomic and educational contexts (1). At the policy level in China, the guarantee for school physical education has also been continuously strengthened. For instance, the Outline for Building a Leading Education Power (2024–2035) explicitly puts forward requirements for the time of comprehensive physical activity for primary and secondary school students, providing an institutional background for the research and practice of health promotion through various forms of sports (Relevant Policy Documents of the Ministry of Education, 2024–2035) (2). Against this background, it is practically important to examine feasible and culturally adaptive forms of sport and their associations with adolescent health indicators.

Physical activity is associated not only with physiological outcomes, such as body weight and cardiorespiratory health, but also with mental health, stress-related experiences, and learning-related indicators. Recent reviews and empirical studies have reported that regular physical activity is associated with lower levels of internalizing problems such as anxiety and depression. Potential explanatory pathways may include sleep, emotional regulation, self-efficacy, and stress responses. However, the strength of causal evidence varies across study designs, and cross-sectional findings should be interpreted as associations rather than causal effects. In addition, academic pressure, sedentary behavior, and insufficient access to resources may reduce adolescents’ participation in physical activity and indirectly affect their mental health (3). Meanwhile, large-sample cross-sectional studies in China have also shown that insufficient physical activity among students is closely related to health cognition, resource supply (e.g., equipment and class hours), and academic pressure, with differences observed among groups such as urban and rural areas, boarding and non-boarding students, and different school types (4). Therefore, further focusing the research on internalizing problem behaviors, which are more concealed but burdensome, and examining physical activity participation in the context of schools and families, will help form an evidence chain with stronger policy and practical orientation.

Most existing evidence on the relationship between physical activity and mental health has focused on general physical activity or aerobic and strength training, whereas relatively less attention has been paid to traditional sports with Chinese cultural and mind–body characteristics, such as Wushu. Wushu practice usually involves physical load, motor control, attention concentration, rule learning, peer interaction, and teacher–student support. These characteristics may provide plausible psychosocial explanations for the observed association between Wushu participation and internalizing problem behaviors. Nevertheless, such mechanisms cannot be directly tested or causally confirmed in a cross-sectional survey. At the same time, adolescents’ participation in physical activity is significantly influenced by factors such as school resources (e.g., venues, equipment, and guaranteed physical education class hours), digital accessibility, and health cognition/values (5, 6). The extent to which Wushu participation is related to mental health indicators may depend not only on participation itself but also on participation frequency, duration, intensity, and the contextual support behind it. Based on this, it is necessary to conduct a more specific quantitative examination of the association between Wushu participation and internalizing problems in Chinese samples.

Based on this background, the present study used cross-sectional baseline survey data to address the following core question: “Is Wushu participation among Chinese adolescents associated with internalizing problem behaviors?” Specifically, the study aims to: (1) describe the distribution characteristics of Wushu participation and internalizing problems under different demographic and school contexts; (2) examine the statistical association between Wushu participation (and its participation characteristics) and internalizing problem behaviors after controlling for confounding factors such as gender, educational stage, region/urban–rural location, boarding status, and school type; (3) discuss potential contextual explanations and intervention implications by combining variables such as school physical education resources and health cognition. This study aims to provide cross-sectional baseline evidence on the association between Wushu participation and internalizing problem behaviors among Chinese adolescents and to generate hypotheses for future longitudinal and intervention studies. It does not aim to evaluate whether Wushu participation causally improves mental health outcomes (7, 8).

### 1.1 Realistic background of physical activity and Wushu participation among children and adolescents

Insufficient physical activity among Chinese adolescents remains an urgent public health issue. Although the policy level has continuously strengthened the supply of school physical education and the guarantee of daily activities, national cross-sectional evidence shows that under the higher standard of daily moderate-to-vigorous physical activity goals, there is still an obvious gap in students’ compliance, with persistent group differences (9). This means that before discussing the mental health effects of a specific sport, it is necessary to acknowledge that “physical activity participation itself” is a health behavior affected by structural conditions, rather than a choice entirely determined by individual willingness.

Under such structural constraints, Wushu has certain contextual adaptability. On the one hand, Wushu can not only be used as part of school physical education curriculum but also carried out through clubs, after-school services, or off-campus training, with strong potential for organized promotion; on the other hand, its dependence on venues and equipment is relatively adjustable, which may be more likely to form regular activity opportunities in some resource-constrained schools. However, whether Wushu can become a stable participation choice is still jointly affected by the “resource-time-cognition” chain, such as the guarantee of physical education class hours, the supply of equipment and teachers, students’ recognition of the value of sports, and the squeeze of disposable time by academic pressure (10).

Furthermore, adolescents’ participation in Wushu is likely to show an unbalanced distribution. Relevant studies have shown that students in rural areas, private schools, boarding students, and those with low frequency of parent–child reunion are more likely to be in a state of insufficient physical activity, and compliance becomes more difficult as the educational stage increases (11). These differences suggest that Wushu participation is not “accessible to everyone”; if the research design does not control for factors such as urban–rural location, educational stage, boarding status, and school type, it is easy to misjudge “people who are more conditionally able to participate are inherently healthier” as “Wushu brings improvement.” Therefore, simultaneously incorporating key contextual variables into cross-sectional baseline research is a basic prerequisite for examining the relationship between Wushu and internalizing problems.

### 1.2 Association between wushu and internalizing problem behaviors

Internalizing problem behaviors are usually manifested as anxiety, depression, withdrawal, and somatization tendencies, and their occurrence and persistence are often intertwined with stress experiences, sleep disturbances, and negative cognitive processing. As a non-pharmacological health promotion method, physical activity is believed to improve mental health through mechanisms such as physiological arousal regulation, emotional release, and social interaction. Relevant studies also emphasize in their discussions that insufficient physical activity is not only associated with physiological risks but also linked to mental health and academic performance, and incorporates academic pressure as an important exposure factor into the model (12). This provides a more school-life-oriented entry point for the reasoning of “Wushu-internalizing problems”: if Wushu can provide stable exercise time and predictable participation rhythm in a high-pressure learning environment, it may play a role in buffering stress.

Compared with general physical fitness activities, Wushu may highlight the continuous process of “skill learning—self-efficacy—emotional regulation.” Routine practice, confrontational training, and motor control require individuals to maintain concentration, continuous feedback, and gradual progress, which easily form a “visible sense of mastery,” thereby improving self-efficacy and self-esteem. If such psychological resources can be accumulated, it can theoretically reduce feelings of helplessness and avoidance, and further be associated with lower levels of internalizing problems. At the same time, organized Wushu training often includes teacher-student interaction, peer collaboration, and standardized behavioral requirements, which may increase social support and a sense of belonging, and this is of potential significance for reducing loneliness and alleviating social withdrawal. The emphasis of relevant studies on the impact of “sports value perception and cognition” on activity behavior also suggests that the psychological benefits of Wushu may be based on value internalization and stable participation (13).

Sleep and daily rhythm may be another key pathway. Regular physical activity helps improve sleep quality, and there is a close connection between improved sleep and reduced internalizing problems. If Wushu training is carried out during school after-school services or fixed club hours, it may not only increase the total amount of activity but also indirectly optimize sleep and emotional states by improving time management and reducing evening screen exposure. It is worth noting that relevant studies suggest that “insufficient access to smart devices” is significantly associated with insufficient physical activity, with differences between urban and rural areas (14); this also means that digital resources may affect the self-monitoring and peer motivation for maintaining physical activity, thereby forming an important contextual boundary condition in the chain of “Wushu participation—sleep—internalizing problems.”

### 1.3 Existing research gaps and the positioning of this study

A large number of studies on adolescents’ physical activity have been accumulated, but there are still problems of “inconsistent standards, crude indicators, and non-specific projects” in the Chinese context. Taking the policy goal of “2 h of daily comprehensive physical activity” as a reference, relevant studies have depicted the current situation of insufficiency in a large national sample, systematically incorporated key factors such as health cognition, value perception, resource supply, physical education class hours, and academic pressure, and revealed the structural pathways affecting activity compliance (10). However, this type of research mainly focuses on overall insufficient physical activity and its health associations, with relatively limited discussion on “whether specific sports are more sensitive to specific psychological outcomes.”

Sports research focusing on mental health also faces two key gaps. First, different sports have obvious differences in intensity structure, skill learning, social interaction, and cultural significance, which may lead to unequal impacts on internalizing problems; as a traditional project, Wushu combines physical fitness and mind–body training characteristics, so it is necessary to conduct a special examination in Chinese adolescent samples. Second, population heterogeneity and selection bias are difficult to avoid. Relevant studies have shown that insufficient activity is more prominent in groups such as rural areas, boarding schools, and private schools, and “cognition and value perception” have stronger explanatory power (15). It can be inferred that if Wushu participants are systematically superior to non-participants in terms of cognition, resources, or time conditions, simply comparing the psychological levels of the two groups may overestimate the effect of Wushu.

Based on the above gaps, this study takes cross-sectional baseline survey as the research framework and conducts an analysis around the statistical association between Wushu participation and internalizing problem behaviors. On the one hand, it fully controls for confounding factors such as gender, educational stage, region and urban–rural location, boarding status, school type, and family support clues in the model; on the other hand, it refines the characteristics of Wushu participation as much as possible at the exposure variable level, expanding from “whether to participate” to dimensions such as frequency, duration, and intensity to be closer to the actual behavioral dosage. At the same time, the study draws on the “resource-time-cognition” explanatory framework proposed by relevant studies, incorporates academic pressure, sports value recognition, physical education resources, and institutional time guarantee into the discussion, so as to provide more operable baseline evidence for the application of school physical education and traditional projects in promoting mental health (16).

## 2 Subjects and methods

### 2.1 Subjects

This study employed a cross-sectional design and analyzed baseline questionnaire data. Following the sampling and implementation procedures outlined by Qin et al. (9) in a national epidemiological survey of adolescent physical activity (random cluster sampling with schools/classes as clustering units for on-site organization), this study was conducted in Guangdong, Jiangsu, Henan, Sichuan, Anhui, and Hainan provinces. Classes served as the basic cluster units for organizing students to complete the questionnaires collectively. A total of 6,642 primary and secondary school students were included. The demographic characteristics of the sample are presented in Table 1: 3,726 boys (56.1%) and 2,916 girls (43.9%). The grade distribution was predominantly high school, with 2,976 students in their third year of high school (44.81%). The age distribution was mainly 16–18 years (63.14%). Regarding residence, 2,562 students resided in urban areas (38.57%), 2,184 in county towns (32.88%), and 1,896 in rural areas (28.55%). The predominant educational attainment for fathers and mothers was “junior high school or below,” accounting for 48.51 and 55.19%, respectively. This study was approved by the Ethics Committee of the School of Physical Education, Suihua University (Approval No. SHU-IRB-2026-008). Written informed consent was obtained from all participants and from parents or legal guardians for minors before the survey.

**Table 1:** Demographic characteristics of the sample.

Variable	Category	Frequency (*n*)	Percentage (%)	Cumulative percentage (%)
Gender	Male	3,726	56.1	56.1
	Female	2,916	43.9	100
Grade	Grades 3–5	786	11.83	11.83
	Grade 7	90	1.36	13.19
	Grade8	660	9.94	23.13
	Grade 9	888	13.37	36.5
	Grade 10	282	4.25	40.74
	Grade 11	960	14.45	55.19
	Grade 12	2,976	44.81	100
Age	9–12 years	810	12.2	12.2
	13–15 years	1,638	24.66	36.86
	16–18 years	4,194	63.14	100
Residence	Urban	2,562	38.57	38.57
	County town	2,184	32.88	71.45
	Rural	1896	28.55	100
Paternal educational level	Junior high school or below	3,222	48.51	48.51
	High school/technical secondary school	1782	26.83	75.34
	Associate/bachelor’s degree	1,260	18.97	94.31
	Graduate degree or above	378	5.69	100
Maternal educational level	Junior high school or below	3,666	55.19	55.19
	High school/technical secondary school	1,632	24.57	79.77
	Associate/bachelor’s degree	1,008	15.18	94.94
	Graduate degree or above	336	5.06	100
Total		6,642	100	100

### 2.2 Research instruments and variable measurement

#### 2.2.1 Martial arts participation scale (revised from PARS-3)

Wushu participation was measured using an adapted version of the Physical Activity Rating Scale-3 (PARS-3) (17). PARS-3 has been widely used in Chinese physical activity research and provides a concise framework for assessing exercise participation through three core dimensions: frequency, duration, and intensity. In the present study, this framework was retained because these three dimensions are consistent with the common understanding of physical activity dose and are suitable for describing adolescents’ regular engagement in Wushu-related activities.

To fit the purpose of this study, the wording of the original PARS-3 items was modified to refer specifically to Wushu and related martial arts activities in school and extracurricular settings, including Tai Chi, Xingyi, Bagua, Shaolin, Changquan, Sanda, Taekwondo, and Karate. The adapted measure included four items: whether the participant had engaged in Wushu or related martial arts activities during the past 12 months; monthly participation frequency; average duration of each session; and perceived exercise intensity. Frequency was assessed on a five-point scale ranging from “less than once per month” to “approximately once daily”; duration was assessed on a four-point scale ranging from “less than 30 min” to “more than 2 h”; and intensity was assessed on a five-point scale ranging from “minimal exertion” to “high intensity with sustained shortness of breath and heavy sweating.” Higher scores indicated higher levels of Wushu participation.

The adaptation was reviewed by two specialists in school physical education and Wushu teaching to ensure that the item wording was understandable and appropriate for primary and secondary school students. Before the formal survey, the questionnaire was checked with a small group of students to confirm clarity of wording and response options. In the present sample, the three graded items of frequency, duration, and intensity showed acceptable internal consistency, with a Cronbach’s *α* of [0.83~0.88]. In addition, the corrected item–total correlations ranged from [0.79~0.84], suggesting that the three items contributed consistently to the composite Wushu participation score. For statistical analysis, the frequency, duration, and intensity items were standardized and averaged to generate a continuous composite score of Wushu participation. Because the adapted instrument has not yet been fully validated as an independent Wushu-specific scale, the score should be interpreted as a self-reported indicator of Wushu participation level rather than as a comprehensive validated measure of Wushu exposure.

#### 2.2.2 Internalizing problems scale (DASS-21)

Internalizing problems were measured using the short form of the Depression, Anxiety, and Stress Scale (DASS-21), referencing the past week (18). The scale consists of 21 observation items across three dimensions: depression, anxiety, and stress, with seven items per dimension. Items are scored on a 4-point Likert scale (0 = never, 1 = sometimes, 2 = often, 3 = almost always). Dimension scores are calculated as the sum (or mean) of the corresponding item scores; higher scores indicate higher levels of negative emotions/stress experiences. To ensure interpretability and consistency with common reporting practices in existing empirical research, this study used the subscale scores for depression, anxiety, and stress as primary psychological outcome variables in the analytical models. The total internalizing problems score was optionally constructed for robustness checks (the method of variable entry is detailed in the Statistical Analysis section). The internal consistency reliability of the DASS-21 in this study was assessed using Cronbach’s *α*, and reliability coefficients for the total scale and each subscale are reported.

### 2.3 Data analysis

Data were analyzed using SPSS version 31.0. Given that the questionnaire variables employed different grading scales, the primary continuous variables were first standardized to reduce systematic bias caused by differences in measurement units. Descriptive statistics were computed to summarize sample characteristics, and Pearson correlation analysis was used to examine the direction and strength of associations among variables. To provide preliminary reliability evidence for the adapted Wushu participation measure, Cronbach’s *α* and corrected item–total correlations were calculated for the three graded participation items: frequency, duration, and intensity. Because the measure was adapted from PARS-3 rather than developed as a new psychometric scale, these analyses were used only as preliminary evidence of internal consistency rather than as full-scale validation.

To assess common method bias, Harman’s single-factor test was employed (19). For inferential analysis, multiple linear regression was used to estimate the cross-sectional associations between Wushu participation and depression, anxiety, and stress. Given the cross-sectional nature of the data, the term “predictor” in the regression models refers to an independent variable statistically associated with the outcome and does not imply temporal prediction or causality. Grade, residence, maternal educational attainment, and variables such as the organizational form of martial arts practice and martial arts type were included as control variables. Multicollinearity diagnostics, including VIF and tolerance, and residual independence tests using the Durbin–Watson statistic were performed. All statistical tests were two-sided, with a significance criterion of *p* < 0.05.

### 2.4 Common method bias testing

To mitigate potential common method bias introduced by self-report data collected at a single time point, Harman’s single-factor test was conducted using unrotated exploratory factor analysis on the main scale items. The results indicated that the first common factor accounted for 26.19% of the total variance, which is below the commonly employed threshold of 40%, suggesting that the risk of common method bias was within an acceptable range.

### 2.5 Descriptive analysis

Table 1 shows that a total of 6,642 valid samples were obtained in this study. The gender distribution was 3,726 boys (56.1%) and 2,916 girls (43.9%). Regarding grade level, grades 3–5 accounted for 11.83%, while the junior high school stage collectively accounted for 24.67% (grade 7: 1.36%, grade 8: 9.94%, grade 9: 13.37%). The senior high school stage had a relatively higher proportion, with grade 12 accounting for 44.81%, grade 11 for 14.45%, and grade 10 for 4.25%. The age distribution was predominantly 16–18 years (63.14%), followed by 13–15 years (24.66%) and 9–12 years (12.20%). In terms of residence, 38.57% lived in urban areas, 32.88% in county towns, and 28.55% in rural areas. Regarding parental educational attainment, the majority of both fathers (48.51%) and mothers (55.19%) had completed “junior high school or below” as their highest level of education, followed by high school/technical secondary school, associate/bachelor’s degree, and graduate degree or above.

### 2.6 Correlation coefficient matrix

Table 2 shows the results of the correlation analysis. Martial arts participation was significantly negatively correlated with depression (*r* = −0.261, *p* < 0.01), anxiety (*r* = −0.251, *p* < 0.01), and stress (*r* = −0.207, *p* < 0.01). Depression, anxiety, and stress were each significantly positively correlated with one another: depression was correlated with anxiety (*r* = 0.462, *p* < 0.01), depression with stress (*r* = 0.423, *p* < 0.01), and anxiety with stress (*r* = 0.473, *p* < 0.01).

**Table 2:** Correlation coefficient matrix.

	M	SD	A	B	C	D
Wushu	2.972	0.832	1			
Depression	2.507	0.555	−0.261**	1		
Anxiety	2.448	0.538	−0.251**	0.462**	1	
Stress	2.508	0.590	−0.207**	0.423**	0.473**	1

*p < 0.05, **p < 0.01, ***p < 0.001.

### 2.7 Regression analysis

Table 3 presents the results of the multiple linear regression analysis. After simultaneously including grade, place of residence, maternal education level, martial arts participation, organizational form, and type, the overall model was significant [*F*(6,6,635) = 54.181, *p* < 0.001], with an *R*^2^ of 0.047 (adjusted *R*^2^ = 0.046) and a Durbin-Watson statistic of 1.907. Although the model reached statistical significance, the proportion of explained variance was limited, indicating that depression among adolescents is likely influenced by additional individual, family, school, and social factors not included in the present model. Specifically, Wushu participation was negatively associated with depression in the adjusted regression model (*B* = −0.003, *β* = −0.191, *t* = −7.079, *p* < 0.001). Grade (*B* = 0.017, *β* = 0.064, *p* < 0.001), place of residence (*B* = 0.098, *β* = 0.143, *p* < 0.001), and maternal education level (*B* = 0.032, *β* = 0.052, *p* < 0.001) were all significant positive predictors. Organizational form (*B* = −0.012, *β* = −0.127, *p* = 0.025) and type (*B* = −0.073, *β* = −0.165, *p* < 0.001) were significant negative predictors. Collinearity diagnostics indicated that the variance inflation factor (VIF) for each predictor ranged from approximately 1.021 to 1.242, suggesting no serious multicollinearity issues.

**Table 3:** Adjusted cross-sectional regression associations between Wushu participation and depression.

Variable	Unstandardized coefficients		Standardized coefficients	*t*	*p*	Collinearity diagnostics	
	*B*	Std. Error	Beta			VIF	Tolerance
Constant	1.367	0.045	–	30.38	0.000**	–	–
Grade	0.017	0.003	0.064	4.883	0.000**	1.213	0.824
Residence	0.098	0.009	0.143	11.149	0.000**	1.147	0.872
Maternal educational level	0.032	0.008	0.052	3.857	0.000**	1.242	0.805
Wushu	−0.003	0	−0.191	−7.079	0.000**	1.162	0.86
Organizational form	−0.012	0.005	−0.127	−2.245	0.025*	1.021	0.979
Category	−0.073	0.015	−0.165	−4.874	0.000**	1.231	0.812
*R* ^2^	0.047						
Adjusted *R*^2^	0.046						
*F*	*F* (6,6,635) = 54.181, *p* = 0.000						
D-W value	1.907						

*p < 0.05, **p < 0.01, ***p < 0.001.

After including grade, place of residence, maternal education level, martial arts participation, organizational form, and type, the overall model was significant [*F*(6,6,635) = 65.632, *p* < 0.001], with an *R*^2^ of 0.056 (adjusted *R*^2^ = 0.055) and a Durbin–Watson statistic of 1.963. This relatively low *R*^2^ suggests that the model captured only a small proportion of the variability in anxiety, and that other unmeasured psychosocial and contextual factors should be considered in future research. Martial arts participation had a significant negative predictive effect on anxiety (*B* = −0.002, *β* = −0.166, *t* = −5.152, *p* < 0.001). Grade (*B* = 0.020, *β* = 0.079, *p* < 0.001), place of residence (*B* = 0.123, *β* = 0.186, *p* < 0.001), and maternal education level (*B* = 0.036, *β* = 0.061, *p* < 0.001) were all significant positive predictors of anxiety. Organizational form (*B* = −0.015, *β* = −0.135, *p* = 0.003) and type (*B* = −0.060, *β* = −0.155, *p* < 0.001) were significant negative predictors of anxiety. Collinearity diagnostics indicated that the variance inflation factor (VIF) ranged from approximately 1.021–1.242, which is within an acceptable range.

After including grade, age, place of residence, martial arts participation, organizational form, and type, the overall model was significant [*F*(6,6,635) = 44.831, *p* < 0.001], with an *R*^2^ of 0.039 (adjusted *R*^2^ = 0.038) and a Durbin–Watson statistic of 1.924. The modest explanatory power of this model indicates that adolescent stress is likely shaped by multiple factors beyond Wushu participation and demographic characteristics, such as academic workload, sleep, family relationships, and school climate. Martial arts participation had a significant negative predictive effect on stress (*B* = −0.002, *β* = −0.156, *t* = −4.364, *p* < 0.001). Grade (*B* = 0.036, *β* = 0.129, *p* < 0.001) and place of residence (*B* = 0.103, *β* = 0.141, *p* < 0.001) were significant positive predictors, whereas age was a significant negative predictor (*B* = −0.085, *β* = −0.102, *p* = 0.001). Organizational form (*B* = −0.020, *β* = −0.143, *p* < 0.001) and type (*B* = −0.070, *β* = −0.159, *p* < 0.001) were also significant negative predictors. Collinearity diagnostics indicated that the VIF values for grade and age were 6.200 and 6.083, respectively (with tolerance values ranging from approximately 0.161–0.164), while the VIF values for the remaining variables were low (ranging from approximately 1.022–1.228).

## 3 Discussion

### 3.1 Differences in the association between martial arts participation and mental health by demographic variables

In this study, Wushu participation remained negatively associated with depression, anxiety, and stress after adjustment for demographic variables, including grade, residence, and maternal educational attainment. This result is consistent with the zero-order correlations reported in Table 2 and suggests that the observed associations were not solely attributable to the selected demographic covariates. The finding is also in line with previous research showing that physical activity participation is generally associated with lower levels of depression, anxiety, and stress among adolescents (20). Nevertheless, because this study used a cross-sectional design, these associations should not be interpreted as evidence that Wushu participation directly prevents or reduces internalizing problems. Reverse causation, selection effects, and residual confounding remain possible. Therefore, the present results should be regarded as baseline evidence of associations between Wushu participation and mental health indicators among Chinese school-aged adolescents.

Regarding demographic variables, grade level was positively associated with all three mental health indicators, suggesting that psychological distress may increase as students advance through school. This pattern is generally consistent with evidence indicating that adolescent development, together with academic pressure, may increase the risk of emotional problems (14). It is also supported by the positive intercorrelations among depression, anxiety, and stress observed in this study, which suggest that psychological distress may cluster and mutually reinforce itself during later school years. Findings on residence-related differences in adolescent mental health have been inconsistent across previous studies, with some reporting higher levels of psychological problems among rural students and others among urban students. These discrepancies may reflect differences in educational stage, resource availability, left-behind or boarding status, and measurement instruments. In the present study, residence was positively associated with all three mental health indicators, suggesting that residence-related factors, such as educational resources, peer environments, and family structure, may be linked to adolescent psychological distress (21). This result should be interpreted cautiously because the direction of the coefficient depends on variable coding. Future studies should use more detailed rural–urban classifications and include family structure and school resource indicators to clarify this association.

Maternal educational attainment was also positively associated with depression, anxiety, and stress in this study. This finding differs from the common expectation that higher parental education is generally associated with better child mental health. Possible explanations include: first, families with higher maternal education may have higher academic expectations and exert greater monitoring over their children, thereby increasing stress and anxiety levels and consequently elevating depression risk (22); second, families with higher education may be more sensitive to their children’s emotional experiences, potentially raising self-reported levels (23); third, the grouping and coding direction of the variables may influence the interpretation of coefficients. This finding, which is not entirely consistent with existing conclusions, suggests that the role of demographic variables is not unidimensional. Future research could introduce mediating or moderating variables such as academic pressure and parent–child relationship quality to explain how educational background translates into risk or protective factors under different family functioning models, and to examine whether martial arts participation buffers the psychological risks associated with high-expectation contexts.

### 3.2 Cross-sectional associations between wushu participation and depression, anxiety, and stress

The regression analyses showed that Wushu participation was negatively associated with depression, anxiety, and stress after adjustment for selected demographic variables. This indicates a stable cross-sectional association in the present sample, but it does not establish whether Wushu participation temporally precedes lower levels of internalizing problems. The regression findings are consistent with the correlation analyses, in which Wushu participation was negatively correlated with depression *r* = −0.261, anxiety *r* = −0.251, and stress *r* = −0.207. This consistency strengthens the internal coherence of the findings. The pattern is also consistent with international and domestic evidence indicating that regular physical activity is associated with lower levels of depression, anxiety, and stress (24). Among adolescents, the negative associations across all three psychological indicators suggest that Wushu participation may be a relevant behavioral correlate of internalizing problems, rather than evidence of a causal buffering effect (14).

When compared with previous studies, the standardized coefficients in this study appeared largest for depression, followed by anxiety and stress. This pattern is generally consistent with literature suggesting that exercise-related associations may be more pronounced for depressive symptoms and relatively smaller or more context-dependent for anxiety and stress (22). One possible explanation is that Wushu participation may be related to behavioral activation, positive experiences, and self-efficacy, whereas stress is often more strongly shaped by external demands, such as examination cycles, homework volume, and sleep deprivation. These demands may be difficult to offset through exercise participation alone (25). This interpretation is also consistent with the positive association between grade level and stress observed in the present study (7), Under conditions of greater time pressure and task demands in higher grades, the relationship between martial arts participation and stress should be understood within a broader school-life context, including time structure management and academic burden.

The relatively low *R*^2^ values across the three regression models should also be noted. Although Wushu participation was statistically associated with depression, anxiety, and stress, the models explained only a small proportion of the variance in these outcomes. Therefore, the statistical significance of the regression coefficients should not be overinterpreted as indicating strong explanatory or predictive power. This finding is consistent with the consensus in the literature that adolescent mental health is shaped by multiple interacting individual, family, school, and broader social factors (26). For example, academic pressure, sleep quality, peer relationships, parent–child relationships, personality traits, socioeconomic status, school climate, and prior mental health conditions may all contribute to depression, anxiety, and stress. In other words, Wushu participation should be understood as one potentially modifiable behavioral correlate of adolescent mental health rather than as a major determining factor. With regard to collinearity diagnostics, the VIF values in Tables 3, 4 are all low, supporting the stability of the estimates. In Table 5, the VIF values for grade and age are elevated, suggesting some collinearity risk among control variables in the stress model. However, the VIF for martial arts participation is low and its coefficient remains significant, indicating that the negative association is unlikely to be driven by spurious effects due to collinearity. Future research could employ longitudinal follow-up or intervention studies to further cross-validate existing causal evidence, and use more refined measures of exercise dose to examine the existence of a dose–response relationship, thereby enhancing the interpretability and generalizability of the conclusions.

**Table 4:** Adjusted cross-sectional regression associations between Wushu participation and anxiety.

	Unstandardized coefficients		Standardized coefficients	*t*	*p*	Collinearity diagnostics	
	*B*	Std. Error	Beta			VIF	Tolerance
Constant	1.207	0.043	–	27.849	0.000**	–	–
Grade	0.02	0.003	0.079	5.987	0.000**	1.213	0.824
Place of residence	0.123	0.008	0.186	14.59	0.000**	1.147	0.872
Maternal education level	0.036	0.008	0.061	4.607	0.000**	1.242	0.805
*W*	−0.002	0.000	−0.166	−5.152	0.000**	1.162	0.86
Organizational form	−0.015	0.005	−0.135	−2.935	0.003**	1.021	0.979
Type	−0.06	0.014	−0.155	−4.17	0.000**	1.231	0.812
*R* ^2^	0.056						
Adjusted *R*^2^	0.055						
*F*	*F* (6,6,635) = 65.632, *p* = 0.000						
D-W value	1.963						

*p < 0.05, **p < 0.01, ***p < 0.001.

**Table 5:** Adjusted cross-sectional regression associations between Wushu participation and stress.

	Unstandardized coefficients		Standardized coefficients	*t*	*p*	Collinearity diagnostics	
	*B*	Std. Error	Beta			VIF	Tolerance
Constant	1.524	0.05	–	30.544	0.000**	–	–
Grade	0.036	0.008	0.129	4.298	0.000**	6.2	0.161
Place of residence	−0.085	0.025	−0.102	−3.421	0.001**	6.083	0.164
Maternal education level	0.103	0.009	0.141	11.484	0.000**	1.044	0.958
*W*	−0.002	0.000	−0.156	−4.364	0.000**	1.117	0.895
Organizational form	−0.02	0.006	−0.143	−3.499	0.000**	1.022	0.978
Type	−0.07	0.016	−0.159	−4.411	0.000**	1.228	0.814
*R* ^2^	0.039						
Adjusted *R*^2^	0.038						
*F*	*F* (6,6,635) = 44.831, *p* = 0.000						
D-W value	1.924						

^*^p < 0.05, ^**^p < 0.01, ^***^p < 0.001.

### 3.3 Cross-sectional associations of organizational forms and wushu event categories with depression, anxiety, and stress

Organizational form and Wushu event category were also associated with depression, anxiety, and stress in the adjusted models. These findings suggest that the context and type of Wushu participation may be relevant to adolescent mental health indicators. However, because organizational form and event category were measured cross-sectionally and were not experimentally assigned, these associations should not be interpreted as evidence that specific organizational modes or Wushu types lead to better mental health outcomes. This result is broadly consistent with evidence that organized physical activity is associated with more favorable mental health indicators than unstructured activity (27). Organized participation may provide regular training routines, peer belonging, coach or teacher support, and rule-based goal feedback, all of which may be related to lower loneliness, higher self-efficacy, and better emotional states (28). Together with the negative association between Wushu participation and internalizing problems observed in this study, the significance of organizational form and event category suggests that the social and structural context of participation may help explain differences in mental health indicators across students (14).

Comparison with existing studies also reveals points worth discussing. Some literature indicates that competitive, hierarchical, or high-intensity training may increase stress and anxiety in certain contexts, particularly when performance orientation and external evaluation are overly emphasized, potentially turning exercise from a “resource” into a “burden.” The negative direction of the coefficients for organizational form and event category in this study appears to differ from the view that some forms of sport participation may impose additional stress. Possible explanations are: first, the measures of organizational form and program type in this study more likely reflect routine, educational, or interest-oriented participation rather than highly competitive, high-stakes training systems; second, the organizational form variable may have simultaneously captured stability and support without distinguishing “supportive organizations” from “pressure-intensive organizations”; third, sources of psychological stress may be predominantly driven by academic structures in higher grades, with any additional stress from sport organizations being relatively diluted. This apparent inconsistency with some sport psychology research suggests that future studies should further differentiate the functional attributes of organizational forms, such as the presence of high-stakes evaluation, the centrality of competitive outcomes, and the extent of punitive training regimes, to avoid conflating disparate organizational ecologies (29).

The findings also show internal consistency: Wushu participation remained associated with mental health indicators after adjustment for other variables, and organizational form and event category were independently associated with these indicators. This suggests that the observed associations may involve both the behavioral dimension of Wushu participation and the contextual dimension of social organization and cultural practice. In light of previous research emphasizing teacher and peer support, value identification, and self-efficacy in adolescent mental health (30), organizational form and event category may be linked to psychological indicators through perceived support, normative structure, and value identification. However, this interpretation remains exploratory and should be tested in longitudinal or intervention studies.

### 3.4 Limitations and future directions

First, the cross-sectional design is the main limitation of this study. Because Wushu participation and mental health outcomes were measured at the same time point, temporal ordering and causality cannot be determined. The observed negative associations may indicate that Wushu participation is linked to lower levels of internalizing problems, but they may also reflect reverse causation, whereby students with better mental health are more likely to participate in Wushu. Unmeasured confounders, such as baseline physical fitness, personality traits, family functioning, academic pressure, school climate, and prior sport experience, may also partly explain the observed associations. Therefore, the findings should not be interpreted as evidence that Wushu participation improves, reduces, or prevents depression, anxiety, or stress. Longitudinal and intervention studies generally provide stronger support for the causal chain in which exercise promotes mental health, whereas cross-sectional results may also reflect reverse causation—that is, students with better mental health may be more willing to participate in martial arts. To achieve stronger cross-validation with existing higher-evidence-level studies, future research should employ multiple time-point follow-ups or quasi-experimental intervention designs (e.g., implementing martial arts courses or clubs in schools) to examine the temporal sequence of changes in mental health indicators as a function of martial arts participation.

Second, the data were collected using self-report questionnaires. Although Harman’s single-factor test suggested that common method bias was within an acceptable range, this test is relatively conservative and cannot rule out systematic errors related to social desirability, recall bias, or same-source measurement. In addition, the Wushu participation measure was adapted from PARS-3 to fit the specific context of Wushu and related martial arts activities. Although the adaptation retained the theoretically relevant dimensions of physical activity dose, namely frequency, duration, and intensity, and although expert review, student clarity checking, Cronbach’s *α*, and corrected item–total correlations provided preliminary support for its content appropriateness and internal consistency, the measure has not yet undergone comprehensive psychometric validation as a Wushu-specific scale. Therefore, the Wushu participation score should be interpreted cautiously as a self-reported behavioral indicator rather than as a fully validated instrument.

Compared with previous studies, the introduction of objective indicators, such as moderate-to-vigorous physical activity duration measured by wearable devices, heart rate load, and sleep duration, together with multi-informant assessments, such as parent/teacher ratings and peer support scales, would facilitate stronger cross-validation with existing objective measurement studies and reduce the risk that observed differences are merely due to measurement methods. Future studies should further examine the construct validity, criterion validity, test–retest reliability, and measurement invariance of Wushu-specific participation instruments across gender, educational stage, and regional groups. Additionally, the elevated VIF values for grade and age in Table 5 indicate collinearity concerns. Future modeling could address this by selecting one of these variables, or by employing centering or stratified variable alternatives, thereby improving estimation stability and aligning with modeling conventions in existing stress research.

Third, the overall explanatory power of the regression models was limited, as indicated by the relatively low *R*^2^ values. This suggests that the variables included in the present study captured only a small proportion of the factors associated with adolescent depression, anxiety, and stress. Although Wushu participation, organizational form, and event category were statistically significant, adolescent mental health is multifactorial and cannot be sufficiently explained by sport participation variables alone. Existing research has shown that academic pressure, sleep quality, peer relationships, parent–child relationships, body image concerns, school climate, socioeconomic status, personality traits, prior mental health status, and access to psychological support may significantly influence depression, anxiety, and stress, and may also interact with exercise participation. Future research could introduce these variables to construct a more comprehensive socio-ecological model and further test potential mediating pathways of martial arts participation, such as self-efficacy, emotion regulation, social support, cultural identity, and discipline. This would help clarify the relative contribution of Wushu participation within the broader system of adolescent mental health determinants. Furthermore, it is recommended that future studies refine the indicators of martial arts dose and type, distinguishing between in-class versus extracurricular, competitive versus recreational, and combat versus routine forms, in order to explain why some studies have found non-significant or inconsistent effects of exercise and to provide a basis for more precise practical implementation.

## 4 Conclusion

Based on this cross-sectional baseline survey of Chinese primary and secondary school students, higher levels of Wushu participation were associated with lower self-reported levels of depression, anxiety, and stress. These associations remained after adjustment for selected demographic covariates. Organizational form of participation and Wushu event category were also associated with mental health indicators, suggesting that the context and type of participation may be relevant to adolescent internalizing problem behaviors. However, because of the cross-sectional design, self-reported measurements, limited covariate adjustment, potential reverse causation, possible selection bias, and the relatively low explanatory power of the models, the findings should be interpreted cautiously. The low *R*^2^ values indicate that many relevant determinants of adolescent mental health were not captured in the present models. Therefore, this study does not establish that Wushu participation causes improvements in mental health or reduces depression, anxiety, and stress. Future longitudinal, quasi-experimental, and intervention studies using validated Wushu-specific measures and more comprehensive confounder control are needed to clarify temporal and causal relationships.

## Data Availability

The original contributions presented in the study are included in the article/supplementary material, further inquiries can be directed to the corresponding author.
